# Recovery of Altered Diabetic Myofibroblast Heterogeneity and Gene Expression Are Associated with CD301b+ Macrophages

**DOI:** 10.3390/biomedicines9121752

**Published:** 2021-11-24

**Authors:** MaryEllen R. Haas, Darlene V. Nguyen, Brett A. Shook

**Affiliations:** 1 Department of Biochemistry and Molecular Medicine, School of Medicine and Health Sciences, The George Washington University, Washington, DC 20037, USA; mehaas@gwu.edu (M.R.H.); darlene@gwu.edu (D.V.N.); 2 Department of Dermatology, School of Medicine and Health Sciences, The George Washington University, Washington, DC 20037, USA

**Keywords:** myofibroblast, macrophage, wound healing, diabetes

## Abstract

Diabetic wound healing is associated with impaired function and reduced numbers of myofibroblasts, a heterogeneous cell population with varying capacities to promote repair. To determine how diabetes alters myofibroblast composition, we performed flow cytometry and spatial tissue analysis of myofibroblast subsets throughout the healing process in diabetic (db/db) and control (db/+) mouse skin. We observed reduced numbers of profibrotic SCA1+; CD34+; CD26+ myofibroblasts in diabetic wounds five days after injury, with decreased expression of fibrosis-associated genes compared to myofibroblasts from db/+ mouse wounds. While the abundance of myofibroblasts remained reduced in db/db mouse wounds compared to controls, the altered myofibroblast heterogeneity and gene expression in diabetic mice was improved seven days after injury. The natural correction of myofibroblast composition and gene expression in db/db wound beds temporally corresponds with a macrophage phenotypic switch. Correlation analysis from individual wound beds revealed that wound healing in control mice is associated with CD206+ macrophages, while the rescued myofibroblast phenotypes in diabetic wounds are correlated with increased CD301b+ macrophage numbers. These data demonstrate how diabetes impacts specific subsets of myofibroblasts and indicate that signaling capable of rescuing impaired diabetic wound healing could be different from signals that regulate wound healing under nonpathological conditions.

## 1. Introduction

Wound healing requires communication between numerous tissue-resident and infiltrating cells [1,2]. Dynamic changes within these signaling networks help regulate three overlapping phases that govern wound healing: inflammation, proliferation, and tissue remodeling. Diabetes is associated with impaired wound healing that is characterized by delayed early activation of proinflammatory immune cells and subsequent persistence of proinflammatory immune cells at later time points after injury [3,4,5,6]. This hinders the progression into the proliferation phase, preventing wound healing from occurring efficiently.

Shortly after the injury, numerous cells produce proinflammatory factors that support the infiltration of inflammatory macrophages and neutrophils [7,8,9]. These myeloid cells clear foreign pathogens and cellular debris and perpetuate inflammation [2,7,10] before activating the initial processes that promote repair [11,12,13]. As inflammation resolves, changes in the gene expression profile of local macrophages support entry into the proliferation phase [10,12,14,15]. Macrophage-derived factors promote the migration and proliferation of keratinocytes, fibroblasts, and endothelial cells to repair the injured tissue [11,13,15,16,17]. Activated fibroblasts, or myofibroblasts, are the leading producers of extracellular matrix (ECM) molecules necessary to strengthen the repaired tissue [18,19,20]. Lineage tracing and cell surface profiling have defined multiple subsets of functionally distinct myofibroblasts in wound beds [21,22,23,24,25]. We have previously shown that myofibroblasts expressing SCA1, CD34, and CD29 are enriched with the potential to generate mature ECM [23]. This population is *Engrailed*-lineage traced and expresses CD26, characteristic of scar-forming fibrogenic myofibroblasts in both mice and humans [22,23,26,27]. This population of myofibroblasts is diminished in wounds of aged mice and undergoes significant age-related changes in its transcriptome [23], underscoring the need to understand how this population is altered under different pathological conditions.

Diabetic wound healing is associated with reduced numbers of alpha-smooth muscle actin (SMA)-expressing myofibroblasts and changes in myofibroblast gene expression that contribute to reduced ECM deposition [28,29,30,31,32]. While impaired diabetic wound healing can result in wounds that persist for months to years in humans [33], repair in diabetic mice is typically delayed, with wounds closing by 14–28 days based on the original size and location of the injury [34,35,36]. Recent reports have begun to characterize functional groups of myofibroblasts that are present during different types of wound healing responses in mice and humans [22,23,37,38,39,40], including multiple groups defining subsets of fibroblasts in human diabetic wounds [39,40]. Though these single endpoint reports provide large datasets that can reveal mechanisms that contribute to impaired healing, additional multi-endpoint studies are needed to define how diabetes alters wound bed myofibroblast composition and function over time and to define mechanisms that support repair in a diabetic state.

In this study, we investigated how diabetes alters myofibroblast heterogeneity and gene expression using diabetic db/db mice. We observed delayed appearance of fibrogenic SCA1+; CD29+; CD26+ myofibroblasts in diabetic mouse wound beds compared to control mice. This altered myofibroblast composition was associated with decreased gene expression of repair- and fibrosis-associated genes in day five wound beds. Surprisingly, seven days after injury, the relative abundance of SCA1+; CD29+; CD26+ myofibroblasts became more prevalent, and diabetes-associated changes in gene expression within this cellular population were not detected. Correlation analyses from individual wound beds revealed that a robust population of CD26- and SMA-expressing myofibroblasts were associated with the emergence of CD206+ macrophages in control mice. However, the diminished disease phenotype was positively correlated with the number of macrophages that express CD301b. Our results demonstrate that myofibroblast heterogeneity and gene expression in diabetic mice bias the wound environment towards a non-reparative state and that the natural correction of the disease phenotype is observed with increased numbers of CD301b+ macrophages that have been shown to support the proliferation of SCA1+; CD29+; CD26+ myofibroblasts in control conditions [23]. These data reveal that the ability of diabetic mice to heal may depend on different factors than what coordinates repair in control animals and highlight the importance of identifying mechanisms that promote tissue repair in a diabetic state.

## 2. Materials and Methods

### 2.1. Mice

Animal care and experiments were performed following the guidance of George Washington University’s Institutional Animal Care and Use Committee (IACUC). Mice of the B6.BKS(D)-*Lepr^db^*/J (Jackson Laboratories, Bar Harbor, ME, USA, Stock #000697; db/db mice) strain with a spontaneous leptin receptor mutation resulting in hyperinsulinemia, hyperglycemia, and obesity were used for these studies, and db/+ littermates were used as controls. During the telogen phase of the hair cycle, male mice (7–9 weeks old) were anesthetized, dorsal fur was shaved, and four full-thickness skin excision wounds were inflicted by biopsy punch (Integra Miltex, Rietheim-Weilheim, Germany), 3–4 mm apart, on either side of the spine between the shoulders and hips as previously described [23,41].

### 2.2. Immunofluorescence and Wound Bed Analysis

After euthanasia, wounds were harvested, embedded in optimal cutting temperature (O.C.T.) compound, frozen, and sectioned using a cryostat (Microm, Boise, ID, USA). To identify the center of wounds, the entirety of the embedded tissue was sectioned from wound edge to wound edge. Sequential tissue sections were distributed across approximately ten slides to allow the center of the wound to be identified on each slide. Tissue cryosections of 16 or 40 μm thickness were fixed with 4% formaldehyde and stained with the following antibodies: CD26 (Abcam, Waltham, MA, USA, 1:250; Novus, 1:50), CD29 (R&D Systems, 1:50), CD45 (Biolegend, 1:500), CD206 (Biolegend, San Diego, CA, USA 1:500), CD301b (eBioscience, San Diego, CA, USA, 1:100), ER-TR7 (Abcam, 1:500), iNOS (Thermofisher, Waltham, MA, USA, 1:100), PU.1 (Cell Signaling, Danvers, MA, USA, 1:100), and SMA (Abcam, 1:400). For histological analysis of each wound, the two central-most wound bed sections were imaged using Zeiss AxioImager M2 (Zeiss, Oberkochen, Germany) with Orca camera (Hamamatsu, Hamamatsu, Japan), analyzed as described below, and averaged together as a single wound bed value. The abundance of SMA+ and ER-TR7+ fibroblasts was calculated using the Color Range feature of Adobe Photoshop to determine the percentage of wound bed pixels occupied by fluorescent staining. ECM proteins were quantified by the previously described corrected total fluorescence calculation [42,43] using ImageJ software (National Institutes of Health, Bethesda, MD, USA). Spatial mapping of fibroblast subsets based on CD45 (negative), CD26, and CD29 expression was performed in MATLAB as previously detailed [23]. Wound bed CD26 immunostaining was assessed by mean gray fluorescence (sum of pixel gray values divided by the total number of pixels selected) of the wound bed within CD45-negative cells using ImageJ. Cell counts were manually tabulated in ImageJ software.

### 2.3. Fluorescence-Activated Cell Sorting Analysis

Intact dorsal mouse skin or wound beds were harvested, minced, and digested using Liberase TM to release single cells. Cells were resuspended in FACS staining buffer (1% BSA in PBS with 2 mM EDTA) and stained with the following antibodies for 30 min on ice: CD45-PE-Cy7 (eBioscience, 1:2000), CD31-APC-Fire750 (Biolegend 1:1000), CD29-Alexa700 (eBioscience, 1:400), CD34-Pacific Blue (BD Bioscience, 1:50), Sca1-V500 (eBioscience, 1:500), CD26-PE-Cy7 (Biolegend, 1:500), CD9-APC (BD Bioscience, 1:100), and CD9-FITC (eBioscience, 1:100). Samples were analyzed on a BD Aria III with FACSDiva software (BD Biosciences, San Jose, CA, USA), and gating and analyses were completed with FlowJo software (BD Biosciences).

### 2.4. RNA Extraction and qRT-PCR

To determine differential gene expression between conditions, FACS-purified myofibroblasts were sorted into DMEM containing 10% FBS. Cells were pelleted by centrifugation. Using TRIzol LS (Invitrogen, Waltham, MA, USA), samples were digested and phase-separated; RNA was extracted in the aqueous phase and purified using the RNeasy Plus Micro Kit (Qiagen, Hilden, Germany). A Superscript III First-Strand Synthesis Kit (Invitrogen) was used to generate cDNA proportionally to the isolated RNA. Gene targets were amplified by qRT-PCR on a LightCycler 480 (Roche, Basel, Switzerland) with SYBR PowerTrack Green master mix (Applied Biosystems, Beverly, MA, USA) and gene-specific primers listed in Supplemental Table S1. Reported results consist of levels of target mRNA normalized to *β-actin*. Data represent the average of biological samples (*n* = 3–4 mice) calculated from technical qPCR triplicates.

### 2.5. Statistics

To determine significance between experimental groups, comparisons were made using Student’s t-test. Analyses across multiple groups were made using a one-way analysis of variance with Bonferroni’s post hoc test using GraphPad Prism for Mac (GraphPad Software, La Jolla, CA, USA) with significance set at *p* < 0.05.

## 3. Results

### 3.1. Diabetic Murine Wound Beds Exhibit Delayed Myofibroblast Population

**Figure 1:**
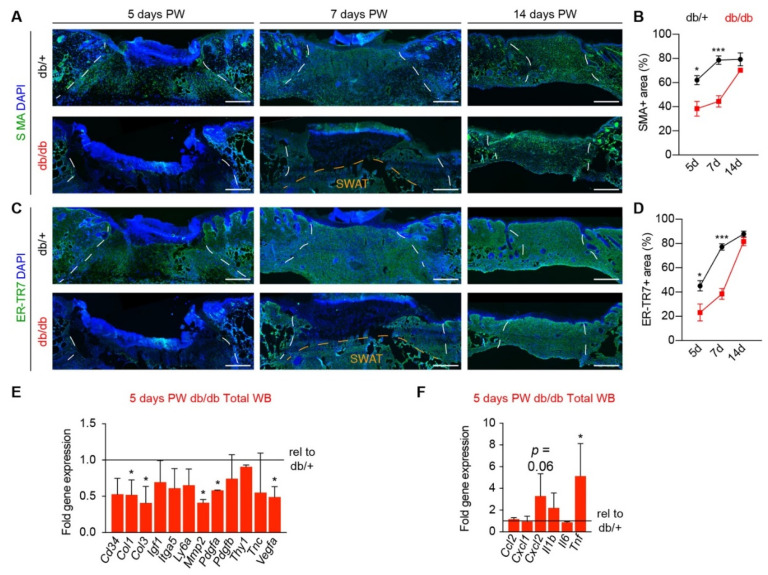
Myofibroblast population is delayed in diabetic mouse skin repair. (**A**–**D**) Sections from the center of wound beds (WB) from db/+ and db/db mice were analyzed for the myofibroblast population at days 5, 7, and 14 post-wounding (PW). (**A**,**B**) Images (**A**) and quantification (**B**) of tissue sections immunostained for SMA. (**C**,**D**) Images (**C**) and quantification (**D**) of tissue sections immunostained for ER-TR7. (**E**) Expression of genes associated with wound healing and fibrosis in db/db wounds compared to db/+ wounds, five days after injury. (**F**) Expression of genes associated with wound inflammation in 5-day PW db/db wounds compared to 5-day PW db/+ wounds. White dashed lines delineate wound edges. Orange dashed lines define the border between the wound bed and subcutaneous white adipose tissue (SWAT). Scale bars, 250 μm. *n* = 4 for each condition. Error bars indicate mean ± SEM. * *p* < 0.05, *** *p* < 0.001.

The rate of skin repair is influenced by many factors, such as hair follicle stage, sex, and type of wound [34,44,45]. To determine how obesity and diabetes associated with db/db mice impact fibroblast/myofibroblast population of wound beds in our model (4 mm full-thickness dorsal skin excision), we harvested wounds from control (db/+) and diabetic (db/db) mice 5, 7, and 14 days post wounding (PW). In control mice, these time points represent periods of robust proliferation and migration of cells that repair the tissue (five days PW), complete repopulation of tissue that results in a hypercellular wound bed (seven days PW), and pruning of the hypercellular wound bed to more closely represent uninjured tissue (14 days PW) [42,46]. Tissue sections from the center of the wounds were immunostained for SMA to label myofibroblasts and ER-TR7 to broadly label fibroblasts. In db/db mice, there was approximately a 50% reduction in the percentage of wound bed area occupied by SMA+ and ER-TR7+ fibroblasts in wound beds five and seven days after injury (Figure 1A–D). This was associated with decreased expression in wound beds of genes associated with fibrosis, such as *Col1*, *Col3*, *Mmp2*, *Pdgfa*, and *Vegfa* (Figure 1E), and higher expression of proinflammatory cytokines such as *Cxcl2* and *Tnf* (Figure 1F) in day five wound beds from db/db mice compared to db/+ mice. Interestingly, there was no detectable difference in the area of wound bed occupied by SMA+ and ER-TR7+ fibroblasts in 14-day wounds (Figure 1A–D), suggesting that the delayed fibroblast population was ameliorated by 14 days after injury in our model.

### 3.2. Fibroblast Heterogeneity Is Altered in Diabetic Wound Beds

**Figure 2:**
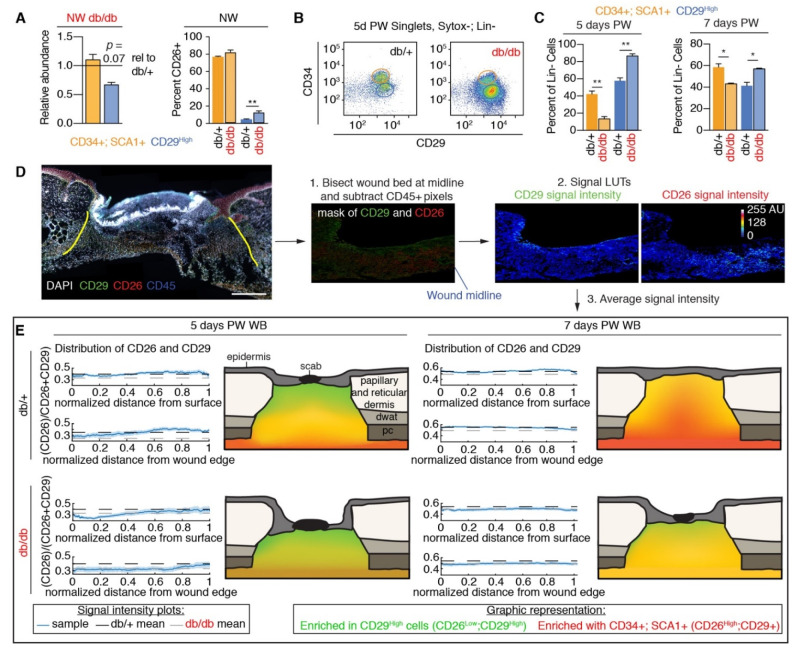
Myofibroblast composition is altered in diabetic mouse wounds. (**A**) Quantification from flow cytometry analysis of the relative abundance of CD34+; SCA1+ and CD29^High^ fibroblasts in db/db uninjured (NW) skin compared to db/+ skin and the percentage that colocalized with CD26. (**B**) Flow plots of CD45-, CD31-lineage negative, Sytox-, single cells from wound beds five days post-wounding (PW) displaying different populations of myofibroblasts based on CD34 and CD29 levels. (**C**) Quantification of the abundance of CD34+; Sca1+ and CD29^High^ myofibroblasts in db/+ and db/db wound beds five days and seven days PW. (**D**) Workflow to spatially quantify CD29 and CD26 signal intensities in wound bed sections immunostained for CD26, CD29, and CD45. (**E**) Quantification and cartoon representations of the average spatial distribution of CD26 and CD29 signals in wound beds five days and seven days after injury in db/+ and db/db mice. Scale bars, 250 μm. *n* = 3–5 for each condition. Error bars indicate mean ± SEM. * *p* < 0.05, ** *p* < 0.01.

While a reduction in proliferation phase myofibroblasts has been documented in wound beds from diabetic mice [28], little is known about how individual subsets of myofibroblasts are altered by diabetes. We have previously described multiple subsets of myofibroblasts that can be identified using cell surface marker expression of CD29, SCA1, and CD34 [23,41]. These myofibroblast subsets are functionally distinct, with different propensities to generate and modify ECM [23]. In particular, a subset of myofibroblasts enriched with CD34, SCA1, and CD26 is associated with efficient wound healing, whereas SCA1-; CD29^High^ myofibroblasts were associated with impaired healing [23]. Using flow cytometry, we investigated if diabetes alters the relative abundance of CD34+; SCA1+, and CD29^High^ mesenchymal cells in the skin and determined if colocalization with profibrotic markers CD9 [47] and CD26 [22] was different in db/db skin compared to db/+ control skin (Figure S1A). We did not observe a statistically significant difference in the relative abundance of CD34+; SCA1+ and CD29^High^ mesenchymal cells in uninjured skin (Figure 2A), and the percentage that colocalized with CD9 was similar in diabetic and control samples (Figure S1B). Interestingly, a greater percentage of CD29^High^ cells colocalized with the profibrotic marker CD26 in uninjured db/db skin versus uninjured db/+ skin (Figure 2A). While these mesenchymal cell populations colocalize with markers of profibrotic fibroblasts, very few cells in intact db/db or control skin express the PU.1 transcription factor that is expressed by activated profibrotic fibroblasts [48] (Figure S1B), suggesting that diabetic skin fibroblasts are not in an active fibrotic transcriptional state. Additionally, inherent differences in gene expression between CD29^High^ and CD34+; SCA1+ fibroblasts are similar in db/db skin compared to what is observed in other control mouse strains [23], such as CD34+; SCA1+ fibroblasts expressing higher levels of *Ccl2* and *Il6* with lower expression of *Il1b* and *Pdgfa*, compared to CD29^High^ fibroblasts (Figure S1C). These data indicate that diabetic skin is not depleted of fibrogenic fibroblasts prior to the injury.

To determine how diabetes alters the composition of wound bed myofibroblasts, we examined the relative abundance of CD34+; SCA1+, and CD29^High^ myofibroblast subsets in wound beds five days post-wounding (PW) in db/+ and db/db mice. Interestingly, wound beds from diabetic mice exhibited a ~25% greater enrichment for CD29^High^ cells and lacked significant enrichment for CD34+; SCA1+ myofibroblasts compared to control mice (Figure 2B,C). These results were similar to what we have previously observed in wounds from aged mice [23], which also are associated with delayed myofibroblast population and impaired wound healing. After the injury, a greater percentage of CD34+; SCA1+ myofibroblasts express CD9 while retaining colocalization with CD26. The percentage of CD34+; SCA1+, and CD29^High^ myofibroblasts that colocalize with CD9 and CD26 was similar in diabetic and control mice, except more CD29^High^ myofibroblasts colocalize with CD26 (Figure S1D), similar to what is observed in uninjured skin (Figure 2A). In line with samples from uninjured skin (Figure S1C) and our previously published data [23], CD34+; SCA1+ and CD29^High^ wound bed myofibroblasts had unique gene expression profiles. For instance, CD34+; SCA1+ myofibroblasts had greater expression of *Fgf7*, *Fn1*, and *Il6*, while CD29^High^ myofibroblasts had greater expression of *Pdgfa* and *Tnc* (Figure S1E), suggesting a functional difference between myofibroblast subsets.

In wild-type strain mice, CD29^High^ myofibroblasts are typically biased to the superficial wound edges [23]. To determine if there are spatial biases to the distribution of CD34+; SCA1+; CD26+ and CD29^High^ myofibroblasts in db/db mouse wound beds, we assessed the distribution of nonimmune (CD45 negative) cells in wound beds based on the expression of CD29 and CD26 in wounds from db/+ and db/db mice (Figure 2D). Similar to wild-type strains, we observed a superficial and wound edge bias of CD29^High^ myofibroblasts in wound beds five days PW (Figure 2E). Interestingly, while there was a delay in CD26-expressing myofibroblasts populating the wound beds of db/db mice, the spatial distribution mirrored that of db/+ at earlier time points (Figure 2E). While there is a clear reduction in the abundance of myofibroblasts seven days PW in wounds of diabetic mice compared to earlier time points in control mice (Figure 1A), there was a surprising similarity in myofibroblast subset distribution and relative abundance between 7-day db/db wound beds and 5-day db/+ wound beds (Figure 2C,E), suggesting that signaling in diabetic wounds begins to promote repair during this time to ameliorate the impaired response.

### 3.3. Corrected Profibrotic Gene Expression Precedes Recovery of Myofibroblast Numbers in Diabetic Wound Beds

**Figure 3:**
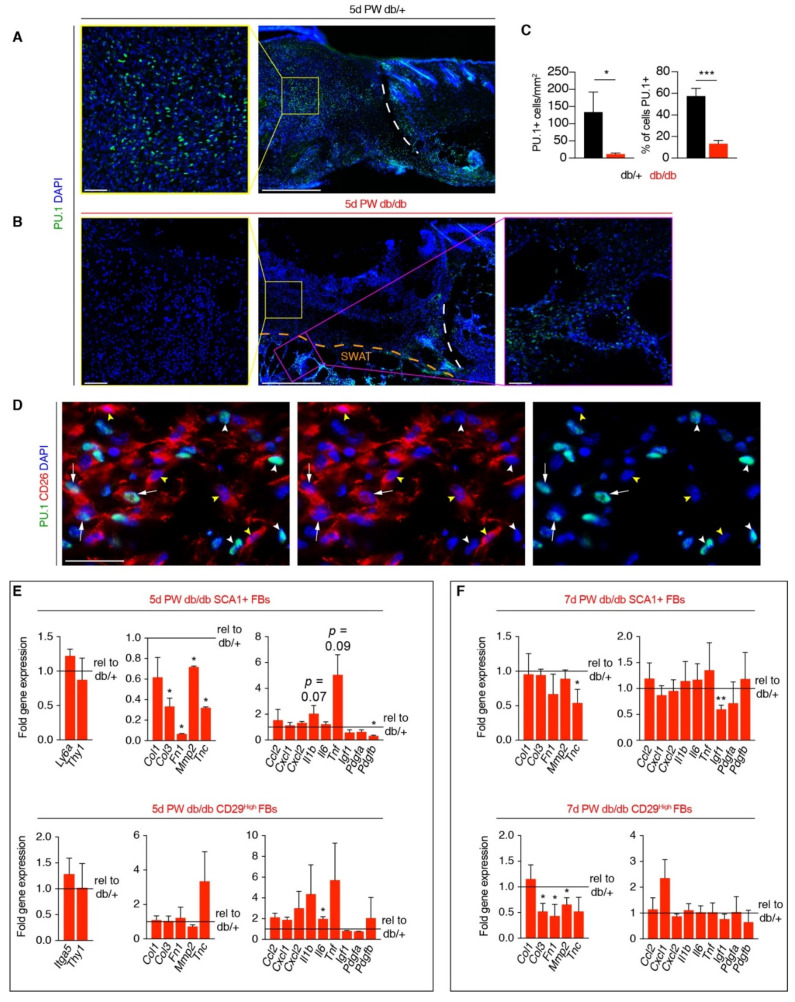
Dysfunctional myofibroblast gene expression is ameliorated during the proliferation phase of repair. (**A**,**B**) Tissue sections from wound beds five days post-wounding (PW) harvested from db/+ (**A**) and db/db mice (**B**) immunostained for PU.1. Scale bars, 250 μm for low magnification images and 75 μm for high magnification images. (**C**) Quantification of the density of PU.1+ cells and percentage of DAPI+ nuclei in tissue sections from db/+ and db/db wound beds five days PW. Arrows indicate CD26+; PU.1+ cells, white arrowheads indicate CD26-; PU.1+ cells, and yellow arrowheads indicate CD26+; PU.1- cells. (**D**) Image from the center of a day five wound bed immunostained for CD26 and PU.1. Scale bars, 75 μm. (**E**,**F**) Quantification of gene expression of CD34; SCA1+ and CD29^High^ myofibroblasts from db/db mice five days (**E**) and seven days (**F**) PW normalized to the same myofibroblast population from db/+ wounds from the same time point. *n* = 3–6 for each condition. Error bars indicate mean ± SEM. * *p* < 0.05, ** *p* < 0.01, *** *p* < 0.001.

Our data support that diabetes is associated with fewer myofibroblasts that have profibrotic potential at both five and seven days after injury. Though surface marker expression is highly predictive of cellular function in control mice, disease-associated changes in gene expression can lead to impaired cellular activation and function [49,50]. To determine if myofibroblasts in 5-day wounds from db/+ and db/db mice are activated to be profibrotic, we quantified the number of wound bed cells that express the transcription factor PU.1. Not only did wound beds from db/db mice have significantly fewer PU.1+ cells compared to db/+ controls, but there was a significant reduction in the percentage of total cells that colocalized with PU.1 (~60% in db/+ vs. 15% in db/db) (Figure 3A–C). While previous reports have implicated CD26-expressing myofibroblasts as drivers of ECM production and scar formation [22], immunofluorescent staining of tissue sections five days PW with CD26 and PU.1 did not reveal strong correlations between CD26 and PU.1 co-expression within the same cells (Figure 3D), suggesting further heterogeneity or different active states may exist within CD26+ myofibroblasts.

To interrogate how diabetes impacts the function of specific myofibroblast subsets, we FACS-isolated CD34+; SCA1+ and CD29^High^ myofibroblasts from wound beds five days PW and examined the expression of genes related to myofibroblast function. We did not observe diabetes-associated changes in gene expression of cellular identity genes, such as *Itga5*, *Ly6a* (SCA1), and *Thy1*; however, diabetes was associated with changes in gene expression of ECM- and inflammation-related genes. Interestingly, gene expression of ECM components, such as *Col1*, *Col3*, *Fn1*, *Mmp2*, and *Tnc*, was significantly reduced in diabetic CD34+; SCA1+ myofibroblasts compared to db/+ controls; however, gene expression in ECM molecules was not significantly altered in CD29^High^ myofibroblasts from db/db mice (Figure 3E). We further explored ECM components through analysis of tissue sections in uninjured skin and wound beds. While tenascin C is not present in uninjured skin, we observed reduced immunofluorescent staining of fibronectin in db/db skin compared to db/+ skin (Figure S2A,B). Interestingly, after injury, the relative intensity of fibronectin staining is similar between db/db and db/+ mice (Figure S2C,D), and tenascin C levels are lower in db/db wounds compared to db/+ controls at both five and seven days PW (Figure S2E,F). These results are in line with our observed changes in gene expression and previous reports demonstrating that diabetes is associated with normal levels of fibronectin [32] but decreased levels of tenascin C after injury.

To further explore how diabetes alters fibroblast function during the proliferation phase, we examined gene expression of ECM molecules and secreted signaling molecules in SCA1+ and CD29^High^ myofibroblasts seven days after injury. Surprisingly, we observed few significant changes in gene expression within the SCA1+ myofibroblast subset (Figure 3F). While we observe reduced ECM molecule expression in CD29^High^ myofibroblasts (Figure 3F), many of these genes are more enriched in CD34; SCA1+ myofibroblasts (Figure S1D) [23]. These data indicate that diabetes-associated changes in gene expression are less pronounced in CD34+; SCA1+ myofibroblasts at the same time post-wounding as when the number of CD34+; SCA1+; CD26+ myofibroblasts increases (Figure 2C,E).

### 3.4. A Macrophage Phenotype Switch Is Concomitant with Changes in Wound Bed Myofibroblasts

**Figure 4:**
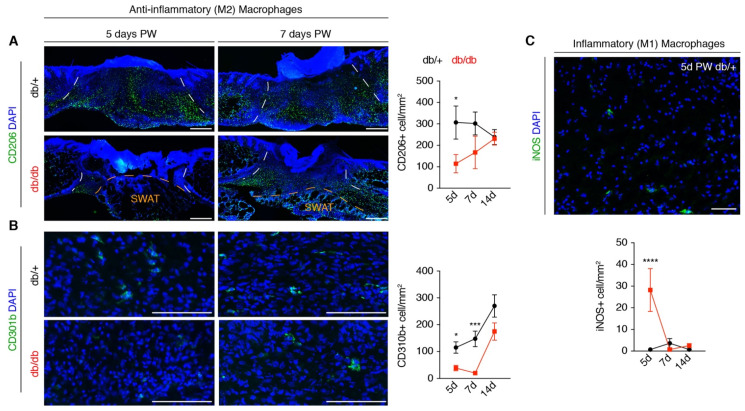
Immune cell phenotypic switch precedes myofibroblast population in db/db wounds. (**A**) Images and quantification from tissue sections from db/+ and db/db wounds 5, 7, and 14 days post-wounding (PW) immunostained for anti-inflammatory macrophage marker CD206. The white dashed lines delineate the wound edges, and the orange dashed line indicates the boundary between the wound bed and deeper subcutaneous white adipose tissue (SWAT). Scale bars, 250 μm. (**B**) Immunostaining images and quantification of CD301b throughout healing in db/+ and db/db wound beds. Scale bars, 100 μm. (**C**) Image from the section of 5-day wound bed immunostained for iNOS and quantification of the number of iNOS+ cells in db/+ and db/db wounds 5, 7, and 14 days PW. Scale bar, 100 μm. *n* = 4–6 for each condition. Error bars indicate mean ± SEM. * *p* < 0.05, *** *p* < 0.001, **** *p* < 0.0001.

Our data reveal a similar gene expression profile in SCA1+ myofibroblasts and a correction of myofibroblast heterogeneity seven days after injury in our wound model (Figure 2C). This indicates that the wound environment of db/db mice is capable of rescuing CD34+; SCA1+ myofibroblast dysfunction prior to the rapid recovery in general myofibroblast population and tissue formation that is observed by two weeks after injury in db/db mice (Figure 1A–D). Macrophage phenotype has been correlated with fibroblast function and wound healing outcomes in humans [40,51,52,53,54], and functional animal studies have confirmed that anti-inflammatory (M2) macrophages support tissue repair [17,46,55,56]. After the injury, iNOS+ inflammatory (M1) macrophages are prevalent during the early phases of repair. As healing proceeds, the number of M1 macrophages decreases as CD206+ M2 macrophages become the predominant phenotype. Over time, CD206+ macrophages begin to express CD301b [46,57]. To define changes in macrophage phenotype that are associated with rescued myofibroblast heterogeneity and function, we quantified the number of CD206+, CD310b+, and iNOS+ macrophages in tissue sections during tissue repair in db/+ and db/db mice (Figure 4A–C). Diabetic mice had fewer CD206+ and CD301b+ M2 macrophages and a greater number of iNOS + M1 macrophages five days PW. Interestingly, the relative numbers of CD206+ and iNOS+ macrophages were not significantly different seven days after injury, corresponding with the timing of rescued SCA1+ myofibroblast gene expression (Figure 4A,C). These data support that a switch in macrophage phenotype is temporally aligned with the recovery of myofibroblast gene expression and subsequent tissue repair. Since these data examine macrophages based general characterization of M1 and M2 subsets, it is important for future lines of research to continue defining how individual macrophage subsets are altered by the diabetic state.

**Figure 5:**
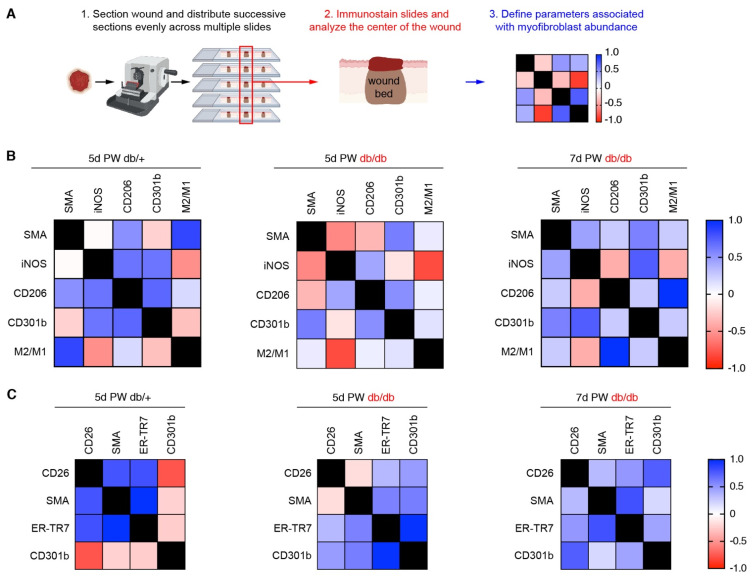
Correlation between macrophage phenotype and myofibroblast population during skin repair. (**A**) Schematic workflow to create tissue repair correlation matrix. Images created with BioRender.com. (**B**,**C**) Correlation matrices from central sections of db/+ and db/db mouse wound beds at days five and seven post-wounding (PW). (**B**) Correlation matrices of SMA abundance with the number of polarized macrophages. M2/M1 ratio was calculated by dividing the number of CD206+ cells/area by the number of iNOS+ cells per area. (**C**) Correlation matrices of the intensity of CD26+ (CD45− cells) and SMA+, ER-TR7+, and CD301b+ cells in wound beds. *n* = 3–6 for each condition.

Our methodology to identify the wound center involves the repeated distribution of subsequent tissue sections onto a set of slides. This generates a set of slides containing tissue sections on each slide that were obtained from different locations throughout the wound bed. On each slide, the central section of the wound can be identified based on the size of the wound bed and various morphological characteristics of epithelial repair (Figure 5A). Slides from the same wound bed can be processed with different antibodies to compare the results between multiple analyses and determine which parameters are correlated (Figure 5A). Using this system, we assessed which immune cell measurements (Figure 4) were associated with the presence of SMA+ wound bed myofibroblasts (Figure 1), including the ratio of CD206+ to iNOS+ macrophages (M2/M1). In day five wound beds from db/+ control mice, the abundance of SMA+ cells was positively correlated with the number of CD206+ macrophages and the ratio of M2/M1 macrophages (Figure 5B). Additionally, there was no correlation with the number of iNOS+ (M1) macrophages and a slight negative correlation with CD301b macrophages (Figure 5B). The slight inverse correlation with CD301b+ macrophages is not surprising, considering most of the wound bed macrophages do not begin to express CD301b until a later time point after injury [46]. These findings are similar to what is observed in various other control mouse strains.

Interestingly, in day five wounds from db/db mice, the amount of SMA+ cells did not correlate with the number of CD206+ macrophages but correlated with the number of CD301b+ macrophages (Figure 5B). The positive correlation of SMA+ cells and CD301b+ macrophages persisted in diabetic wounds seven days after injury (Figure 5B). These data suggest that the cells and possibly the signaling pathways required to promote wound healing in diabetic mice are different from those found in control animals. We further investigated the connection between CD301b+ macrophages and wound bed myofibroblast populations by looking at the association of CD26 intensity (data from Figure 2) and SMA and ER-TR7 cells abundance (Figure 1A,B) with CD301b+ macrophages. In day five wounds from db/+ mice, CD26, SMA, and ER-TR7 levels all positively correlated with each other, but these measurements were inversely correlated with the number of CD301b+ macrophages (Figure 5C). Surprisingly, the amount of CD26+ mesenchymal cells in wound beds did not correlate with the number of SMA+ cells in day five wounds from db/db mice (Figure 5C). Nevertheless, the abundance of CD26+, SMA+, and ER-TR7+ cells all positively correlated with the abundance of CD301b+ macrophages (Figure 5C). By seven days PW, the relative abundance of CD26+ cells in db/db mouse wound beds positively correlated with SMA+, ER-TR7+ and CD301b+ cell numbers (Figure 5C). These data provide a disease context to strengthen the previously published connection between CD301b+ macrophages and fibrogenic CD26+ myofibroblasts [23].

## 4. Discussion

Progression of tissue repair into the proliferation phase has been linked to a switch in macrophage polarization [10,40,46,51], as anti-inflammatory macrophages initiate repair. Here, we revealed that diabetic mouse wounds have both fewer myofibroblasts with fibrogenic capacity and impaired gene expression within myofibroblast subsets. Collectively, these changes contribute to diminished ECM production during diabetic wound healing [32,58]. Our 4 mm biopsy punch model of dorsal skin excision enables more rapid recovery of wound healing defects than larger wound paradigms. This allowed us to capture a time point in diabetic wound healing when myofibroblast numbers are still reduced compared to controls; however, the relative abundance of myofibroblast subsets and repair-related gene expression within each subset are not significantly different. Correlation analysis revealed that efficient wound healing in control mice is associated with CD206-expressing anti-inflammatory (M2) macrophages. This same analysis identified that the ability of diabetic mouse wounds to rescue the impaired healing phenotype is not associated with CD206+ macrophages but with CD301b-expressing macrophages. These macrophages have been shown to be important for the expansion of fibrogenic wound myofibroblasts and efficient wound healing [23,46]. These findings are supported by animal studies that demonstrated that amelioration of impaired healing could occur through administration of (1) factors that rescue defects in early macrophage infiltration [16,59,60,61] or (2) factors derived from anti-inflammatory macrophages that promote repair [23,62,63,64,65,66,67]. These data not only describe how diabetes alters myofibroblast composition and gene expression but also differences in how wound healing in control and diabetic animals may rely on subtle differences in anti-inflammatory macrophage subsets to support tissue repair.

Recently, single-cell RNA-sequencing has been used to define the cellular composition in human diabetic skin and wound samples [39,40,68]. These studies provide tremendous insights into cellular heterogeneity and changes in gene expression between conditions; however, additional time points throughout the healing process are needed to fully understand how the environment changes over time. This challenge is compounded by the inability to identify a chronic or nonhealing wound patient until months after the injury [33]. At this late time point after injury, the cellular and molecular interactions dictating local cellular responses are different than what is observed during normal acute repair [28]. Using a 4 mm dorsal skin excision wound model, we examined repair at multiple time points during the proliferation phase to define how myofibroblast heterogeneity is altered in db/db mice compared to db/+ control animals. The lack of CD26+; SCA1+ fibroblasts early during the proliferation phase is consistent with what is observed with age [23,69]. Later during the proliferation phase, CD26+; SCA1+ myofibroblasts become more abundant than other myofibroblast subsets in diabetic wounds. This change is observed alongside a recovery in impaired myofibroblast gene expression but prior to a broader rescue of total myofibroblast numbers, suggesting that the wound environment can rescue impaired healing in diabetic mice. Performing transcriptomics at multiple time points throughout the healing process would allow future studies to explore mechanisms necessary to overcome the diabetic environment and contribute to new therapeutic approaches.

Analyzing multiple parameters in tissue sections from the center of the same wound beds allowed us to determine cellular events associated with improved outcomes at multiple time points. This comprehensive correlation analysis revealed that diabetic wounds with greater myofibroblast abundance, including more fibrogenic CD26+ myofibroblasts, correlated with greater numbers of CD301b+ macrophages at both five and seven days after injury. While this is in line with other reports [23,26,46,70], the correlation was not detectable in our data that averaged numbers within a group, a surprising result, given that CD301b+ cell numbers were low in 5- and 7-day wound beds from db/db mice with minimal variation between animals. This result reveals that averaging data groups is susceptible to losing valuable data based on the variation within a group. Additionally, our correlation analyses revealed that different M2 macrophage populations were associated with improved myofibroblast numbers in diabetic versus control groups. Recently, increased attention has been given to experiments aimed at defining mechanisms that support diabetic healing in humans [39,40]. Researchers have started to rely less on data from lean individuals and more on samples from diabetic healers as controls for studies examining impaired diabetic wound healing [5,39]. Our correlation analyses capitalized on the variation that occurs during diabetic wound healing in mice to reveal conditions associated with improved myofibroblast repopulation relative to wounds with more myofibroblast dysfunction. Additional studies will be needed to determine if CD301b+ macrophages functionally contribute to the corrected myofibroblast phenotypes during delayed diabetic wound healing.

## 5. Conclusions

In summary, while murine diabetic wound healing is associated with fewer profibrotic myofibroblasts and decreased expression of genes associated with the repair, diabetic wounds that can heal have greater numbers of CD301b-expressing macrophages. It will be important for future investigations to determine how factors influence the ability of myofibroblasts to recovery, especially since the length of diabetes, size of the wound, or animal species can impact healing rates [34,44,45,71]. Additionally, it will be critical to determine if the same cellular and molecular mechanisms are responsible for the recovery in different injury paradigms since slight changes in wound size can substantially impact the contribution from specific fibroblast lineages and regenerative potential of the injury response [24,37,41,72,73]. Since diabetic human fibroblasts undergo epigenetic changes that are not easily rescued in culture [74], future studies are needed that explore differences in diabetic healers and diabetic non-healers. Future human studies will be critical for uncovering clinically relevant mechanisms that promote myofibroblast function and tissue repair in diabetics.

## Data Availability

The data that support the findings of this study are available from the corresponding author upon reasonable request.

## Supplementary Materials

The following are available online at https://www.mdpi.com/article/10.3390/biomedicines9121752/s1, Figure S1: Characteristics of wound bed myofibroblast populations in diabetic mouse skin and wound beds. Figure S2: ECM composition is altered in diabetic mouse skin and wound beds. Table S1: qPCR Primers.
