# Supplementary material for: Recovery of Altered Diabetic Myofibroblast Heterogeneity and Gene Expression Are Associated with CD301b+ Macrophages
Source: Biomedicines. 2021 Nov 24;9(12):1752. doi: 10.3390/biomedicines9121752 (PMC8698992; doi:10.3390/biomedicines9121752)
Supplement: Supplementary file 1 [file biomedicines-09-01752-s001.zip › biomedicines-1432995-supplementary.pdf]

biomedicines

MDPI

Article

Recovery of Altered Diabetic Myofibroblast Heterogeneity and Gene Expression Associated with CD301b+ Macrophages

MaryEllen R. Haas [1], Darlene V. Nguyen [1] and Brett A. Shook [1,2,*]

[1] Department of Biochemistry and Molecular Medicine, School of Medicine and Health Sciences, The George Washington University, Washington, DC 20037, USA; mehaas@gwu.edu (M.R.H.); darlene@gwu.edu (D.V.N.)
[2] Department of Dermatology, School of Medicine and Health Sciences, The George Washington University, Washington, DC 20037, USA
* Correspondence: brettshook@gwu.edu

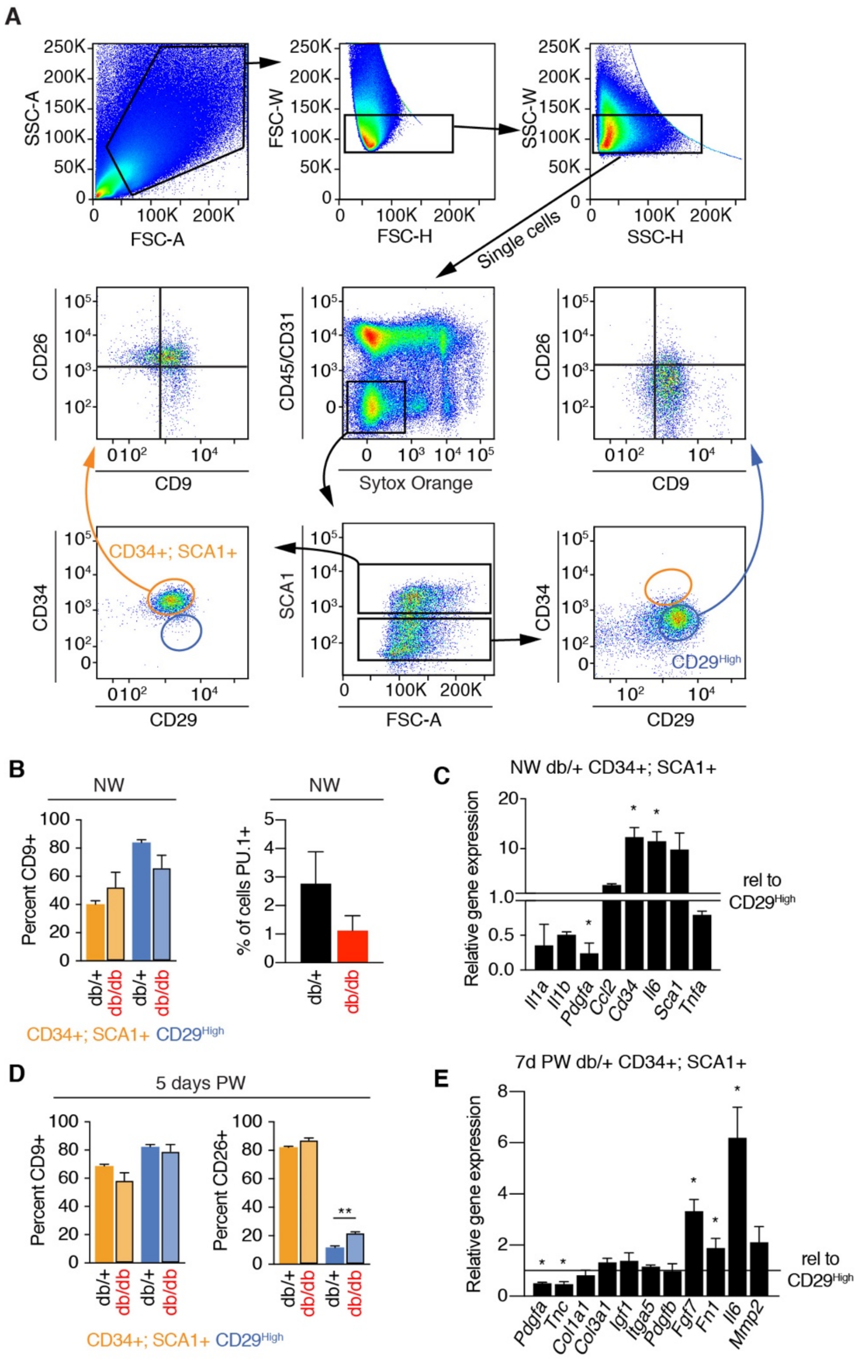

Figure S1. Characteristics of mesenchymal cell subsets in diabetic mouse skin and wound beds. **(A)** Gating strategy to define mesenchymal cell populations in mouse skin and wound beds. **(B)** Quantification of CD9+ from flow cytometry analyzed CD34+; SCA1+ and $CD29^{High}$ cells and quantification of PU.1+ cells in immunostained tissue sections from non-wounded (NW) db/+ and db/db mouse skin. **(C)** Quantitative RT-PCR analysis of genes that characterize CD34+; SCA1+ and $CD29^{High}$ mesenchymal cell subsets in uninjured skin. Results show the expression levels in CD34+; SCA1+ cells relative to $CD29^{High}$ mesenchymal cells. **(D)** Flow cytometry quantification of CD9+ and CD26+ colocalization in myofibroblast subsets from db/+ and db/db mouse skin wounds isolated 5-days post-wounding (PW). **(E)** Quantitative RT-PCR analysis of genes that characterize CD34+; SCA1+ and $CD29^{High}$ myofibroblast subsets. Cell populations were isolated from wound beds 7-days PW. Results show the expression levels in CD34+; SCA1+ myofibroblasts relative to $CD29^{High}$ myofibroblasts. n = 3-5 for each condition. Error bars indicate mean ± SEM. *$p < 0.05$.

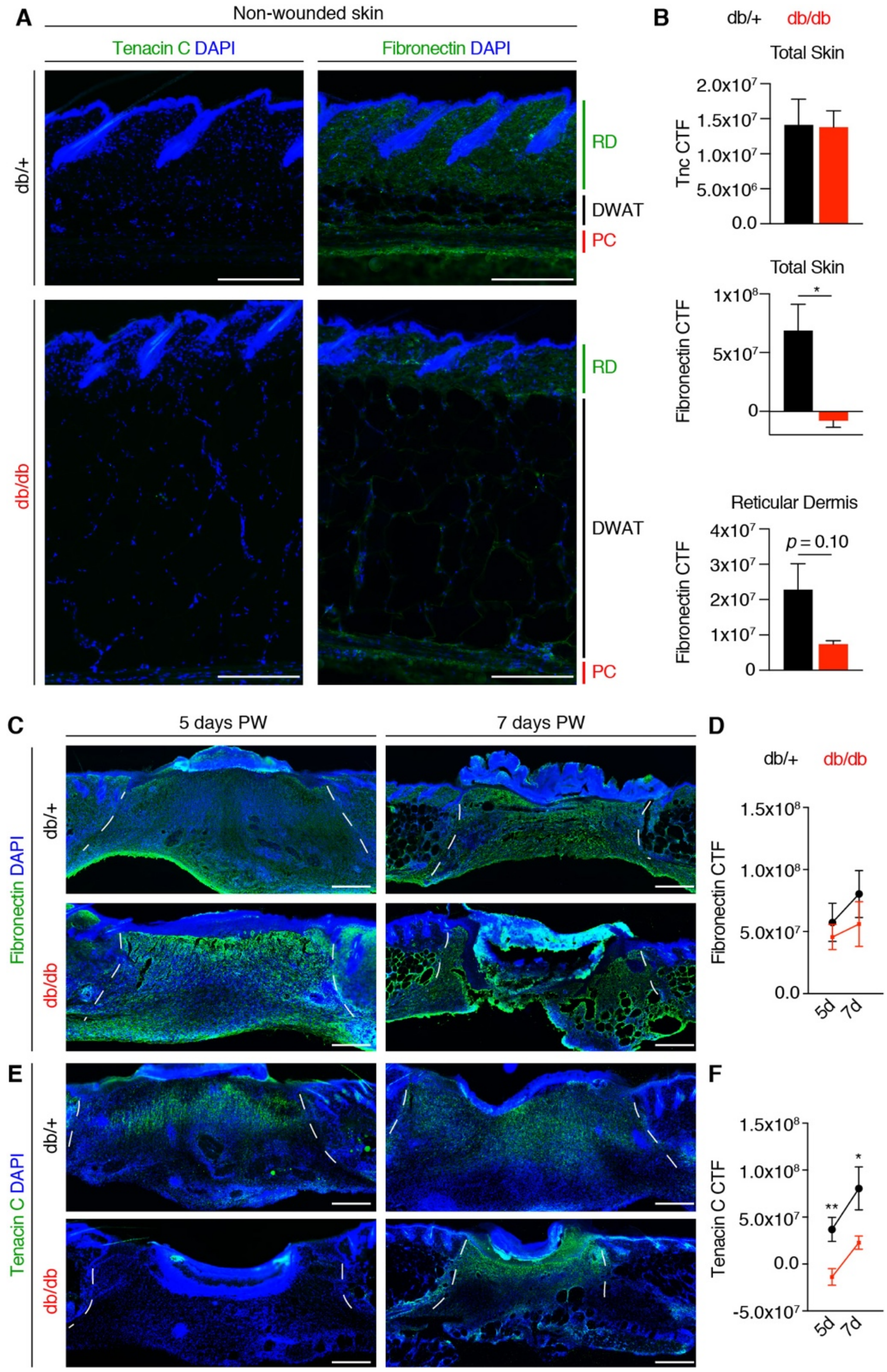

Figure S2. ECM composition is altered in diabetic mouse skin and wound beds. **(A)** Sections from db/+ and db/db mouse skin were immunostained for tenacin C and fibronectin. **(B)** Quantification of relative fluorescent intensity of tenacin C and fibronectin in uninjured skin. **(C-D)** Images from tissue sections were immunostained for fibronectin **(C)** and the relative intensity of the signal was quantified **(D)** in wound beds 5- and 7-days post-wounding (PW). **(E-F)** Images from tissue sections were immunostained for tenacin C **(E)** and the relative intensity of the signal was quantified **(F)** in wound beds 5- and 7-days post-wounding (PW). White dashed lines delineate wound edges. Scale bars, 250µm. n = 3-6 for each condition. Error bars indicate mean ± SEM. *$p < 0.05$, **$p < 0.01$. CTF, corrected total fluorescence.
